# ST6Gal1: Oncogenic signaling pathways and targets

**DOI:** 10.3389/fmolb.2022.962908

**Published:** 2022-08-29

**Authors:** Sajina GC, Susan L. Bellis, Anita B. Hjelmeland

**Affiliations:** Department of Cell, Developmental and Integrative Biology, University of Alabama at Birmingham, Birmingham, AL, United States

**Keywords:** ST6GAL1, cancer, pathways affecting cancer, targets, sialyltransferase, sialylation

## Abstract

The Golgi-sialyltransferase ST6Gal1 (βgalactosidase α2,6 sialyltransferase 1), adds the negatively charged sugar, sialic acid, to the terminal galactose of N-glycosylated proteins. Upregulation of ST6Gal1 is observed in many malignancies, and a large body of research has determined that ST6Gal1-mediated α2,6 sialylation impacts cancer hallmarks. ST6Gal1 affects oncogenic behaviors including sustained proliferation, enhanced self-renewal, epithelial-to-mesenchymal transition, invasion, and chemoresistance. However, there are relatively few ST6GaL1 related signaling pathways that are well-established to mediate these biologies: greater delineation of specific targets and signaling mechanisms that are orchestrated by ST6Gal1 is needed. The aim of this review is to provide a summary of our current understanding of select oncogenic signaling pathways and targets affected by ST6Gal1.

## Introduction

Since abnormal glycosylation in malignancy was first described in 1969, it has been established to affect cancer hallmarks (Meezan et al., 1969; Pinho and Reis, 2015; Munkley and Elliott, 2016; Vajaria and Patel, 2017). Interestingly, a large proportion of cancer biomarkers approved by the FDA are glycosylated proteins, further suggesting the importance of glycobiology in cancer (Moss et al., 2005; Gilgunn et al., 2013; Thomas et al., 2021). One of the glycan changes consistently observed in cancer is hyper-sialylation (Bull et al., 2014; Rodriguez et al., 2018; Li and Ding, 2019; Xu et al., 2021). Sialylation is the process in which sialic acid is added to the terminal end of glycoproteins and glycolipids by enzymes called sialyltransferases. Amongst more than 20 sialyltransferases found in the human body (Harduin-Lepers et al., 2001; Hugonnet et al., 2021), growing evidence has illustrated elevated expression of the Golgi β-Galactoside α-2,6-Sialyltransferase 1 (ST6Gal1) in various malignancies including, but not limited to, breast cancer (Lu et al., 2014), cervical cancer (Wang et al., 2003), ovarian cancer (Wichert et al., 2018), prostate cancer (Wei et al., 2016), pancreatic cancer (Schultz et al., 2016), colon cancer (Swindall and Bellis, 2011), gastric cancer (Gretschel et al., 2003), leukemia (Mondal et al., 2010; Zhang et al., 2022) hepatocellular carcinoma (Chen et al., 2000) and melanoma (Agrawal et al., 2017). This elevation of ST6Gal1 in cancers is often attributed to gene amplification (Dorsett et al., 2021). However, ST6Gal1 is regulated in neoplastic development through multiple mechanisms that include transcription factors (HNF1, Sox2, SP1) (Svensson et al., 1992; Taniguchi et al., 1998; Xu et al., 2003; Milflores-Flores et al., 2012; Dorsett et al., 2019), epigenetic factors (gene methylation, miR9, miR-213-3p. mir-200) (Schliekelman et al., 2011; Minami et al., 2013; Antony et al., 2014; Fleischer et al., 2014; Kroes and Moskal, 2016; Vojta et al., 2016; Han et al., 2018; Tao et al., 2019) as well as post-transcriptional and posttranslational modifications like cleavage by BACE1-β secretase (Chen et al., 2000; Kitazume et al., 2001; Lee et al., 2012; Isaji et al., 2014; Bhide and Colley, 2017; Welch and Munro, 2019; Krick et al., 2021).

**FIGURE 1:**
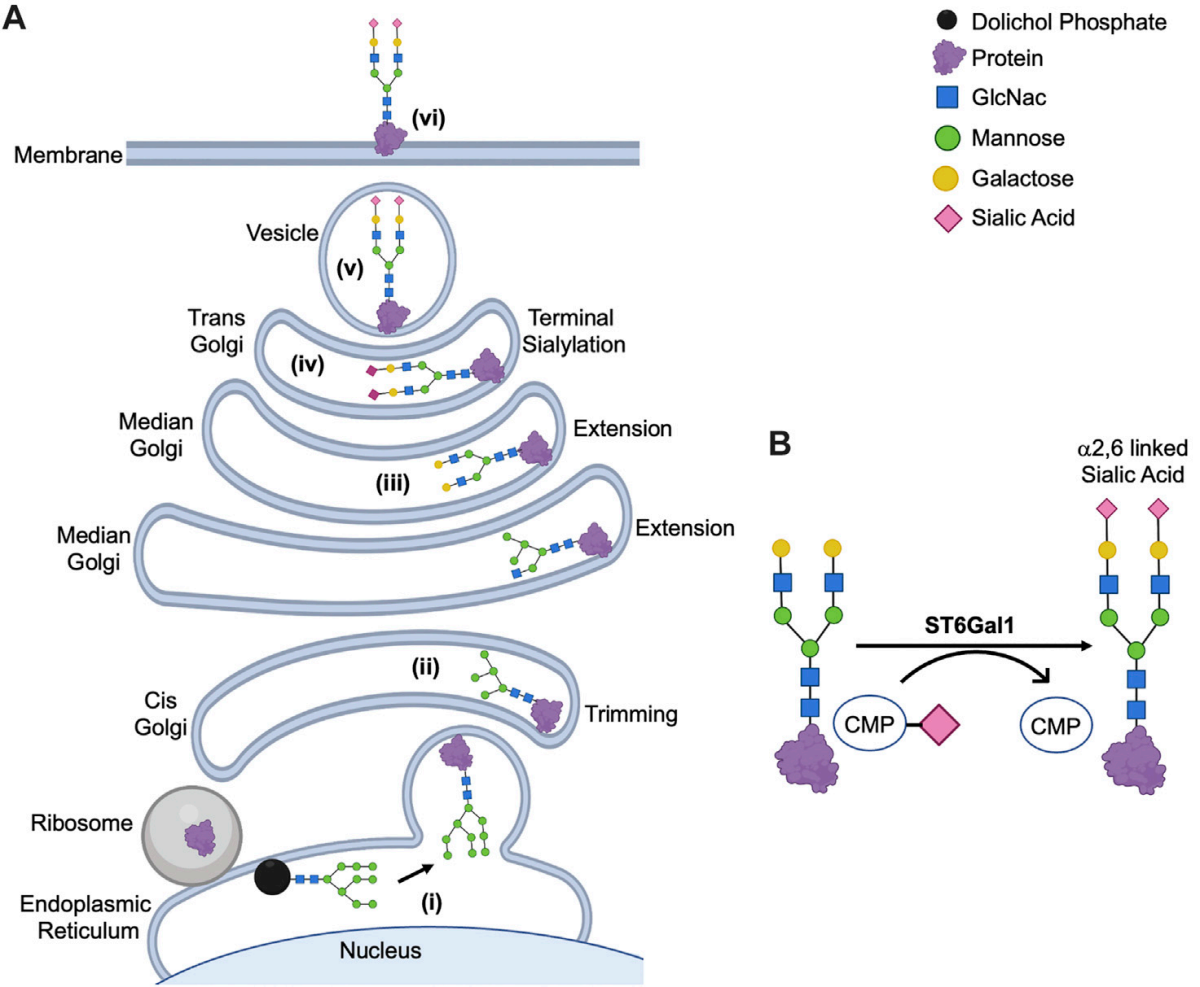
Posttranslational Modification of N-Glycoproteins in Golgi. **(A)** Mature glycan linked to dolichol phosphate is added to the protein synthesized in rough endoplasmic reticulum (i). The glycoproteins are modified *via* trimming in cis-Golgi (ii), extension in median Golgi (iii) and terminal sialylation by ST6Gal1 among other sialyltransferases in trans-Golgi (iv). The mature proteins are transported in vesicles (v) to the membrane (vi). **(B)** ST6Gal1 adds α2,6 linked terminal sialic acid to the N-linked glycoproteins using CMP-Sialic acid as a donor. The schematic diagram was created using Biorender (https://biorender.com).

**FIGURE 2:**
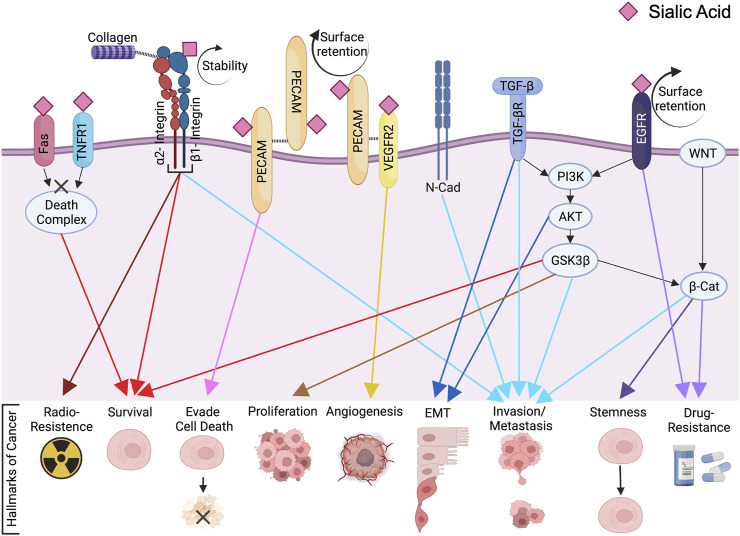
ST6Gal1-mediated Signaling Cascades in Cancer. The membrane bound N-glycoproteins synthesized and processed in secretory pathways (including Fas, TNFR1, β1-integrin, PECAM, VEGFR2 and EGFR among others) are α2,6 sialylated by ST6Gal1. This can affect protein cell surface retention, stability, clustering and/or activation. Through direct and indirect signaling pathways, ST6Gal1 promotes oncogenic characteristics in many cancers. The pink diamond represents α2,6 linked sialic acid, although the full glycan structure is not shown for simplification. The figure was created using Biorender (https://biorender.com).

ST6Gal1 adds sialic acid to the terminal galactose of N-glycoproteins in an α2,6 bond in the trans-Golgi (Figure 1). This α2,6 sialylation by ST6Gal1 is not only a prognostic marker for select cancers but also a driver of malignant progression (Munkley and Elliott, 2016). Mounting reports have implicated ST6Gal1 in eliciting tumorigenic processes like sustained proliferative signaling (Zhao et al., 2014; Wichert et al., 2018), evasion of growth suppressors (Garnham et al., 2019), resistance to cell death (Peter et al., 1995; Meesmann et al., 2010; Liu et al., 2011a; Suzuki et al., 2015), enabling replicative immortality (Garnham et al., 2019), activation of invasion (metastasis) (Wang et al., 2003; Lu et al., 2014; Zhao et al., 2014; Wei et al., 2016), promoting angiogenesis (Croci et al., 2014a; Meng et al., 2015), deregulating cellular energetics (Hsieh et al., 2017) and immune evasion (Hennet et al., 1998; Engdahl et al., 2018) (Figure 2). While there is much to be investigated about phenotypic effects of ST6Gal1 in cancers, even more information is needed on the ST6Gal1-mediated signals that lead to protumorigenic cellular behaviors. Nonetheless, some critical oncogenic pathways involving PI3K/AKT, Wnt/β-catenin and targets for ST6Gal1-mediated sialylation like Epidermal Growth Factor Receptor (EGFR), Platelet and Endothelial Cell Adhesion Molecule (PECAM), Tumor Necrosis Factor Receptor (TNFR), and Vascular Endothelial Growth Factor Receptor (VEGFR) have been identified (Figure 2; Table 1). The purpose of this review is to provide succinct insight into the currently elucidated major targets of, and molecular mechanisms mediated by, ST6Gal1 that contribute to cancer progression.

**TABLE 1:** Pathways and targets affected by ST6Gal1 to impact cancer hallmarks.

Pathway/ Target	α2,6 Sialylation		Cancer Characteristics		Cancer/ Cell Type	References
	Status	Affect	Hallmark	Effect		
H-Ras^V12G37^/ RalGEF			EMT	Promotion	Colon Cancer	Dalziel et al. (2004)
PI3K/AKT/GSK3β/-catenin			Proliferation, Invasion, Metastasis	Promotion	Prostate Cancer	(Romorini et al., 2016; Wei et al., 2016)
PI3K/AKT			EMT, Invasion, Metastasis	Promotion	Ovarian, Breast	(Isaji et al., 2014; Lu et al., 2014)
HER2-AKT-ERK			Chemo-resistance	Promotion	Gastric cancer	Liu et al. (2018)
Wnt/β-catenin			Cancer Stemness, Chemo-resistance	Promotion	Pancreatic cancer, CRC	(Cui et al., 2018; Britain et al., 2021; Chakraborty et al., 2022)
TGF-β			EMT, Invasion, Metastasis	Promotion	Breast cancer	Lu et al. (2014)
β1-Integrin	Yes	Clustering and Stability	Radio-resistance	Promotion	Colon Cancer	Lee et al. (2008)
β1-Integrin/ paxillin/AKT			Radio-resistance	Promotion	Colon cancer	Lee et al. (2010)
α5β1 Integrin/FAK			Invasion, Metastasis	Promotion	HCC	Han et al. (2018)
α5β1 Integrin			Invasion, Metastasis	Promotion	HCC	(Yu et al., 2013; Chen et al., 2021)
PECAM	Yes	Surface retention, clustering	Invasion	Promotion	mouse endothelial cells	(Kitazume et al., 2014; Lee et al., 2014)
PECAM/VEGFR2/ β3-Intergrin			Angiogenesis	Promotion	Lewis lung carcinoma	Imamaki et al. (2018)
VEGF			Angiogenesis	Promotion	Osteosarcoma	Meng et al. (2015)
HIF-1			Angiogenesis	Promotion	Ovarian, pancreatic cancers	Jones et al. (2018)
Fas	Yes	Internalization	Evading Cell Death	Inhibition	Colon Cancer	(Peter et al., 1995; Keppler et al., 1999; Swindall and Bellis, 2011; Swindall et al., 2013; Suzuki et al., 2003)
TNF			Evading Cell Death	Inhibition	Gastric cancer	Alexander et al. (2020)
TNFR1	Yes	Internalization	Evading Cell Death	Inhibition	Pancreatic and ovarian cancer	Holdbrooks et al. (2018)
EGFR	Yes	Dimerization	Invasion	Promotion	Lung cancer	Liu et al. (2011b)
EGFR			EMT, Invasion, Metastasis	Promotion	Pancreatic Cancer	Britain et al. (2021)
EGFR			Chemo-resistance	Promotion	Colon, ovarian cancers	(Park et al., 2012; Britain et al., 2018)
FGFR1-ERK/FAK			Invasion, Metastasis, Chemo-resistance	Promotion	Ovarian cancer	Ou et al. (2020)
Notch/Hes1/MMPs			Invasion, Metastasis	Promotion	NSCLC	Yuan et al. (2018)
CD147/MMPs			Immune Evasion	Promotion	HCC	Wang et al. (2019)

## ST6Gal1 and neoplastic pathways

### RAS signaling

Activating mutations in the family of RAS proteins (KRAS, NRAS, and HRAS) have been well described to cause oncogenic transformation (Hobbs et al., 2016; Gimple and Wang, 2019). The RAS/RAF/MEK/ERK signaling cascade is highly implicated in oncogenic transcription leading to cell cycle progression, and the RAS/PI3K/AKT pathway induces malignant characteristics like cell survival, growth, and metabolic shifts (Gimple and Wang, 2019). More than 3 decades ago, ST6Gal1 was found to be elevated with overexpression of N-Ras in NIH 3T3 fibroblasts and c-HA-Ras in FR3T3 fibroblasts (Easton et al., 1991; Le Marer et al., 1992; Vandamme et al., 1992). Seales et al. (2003), later verified these findings in the HD3 colon epithelial cell model (Seales et al., 2003). Further, in 2004, Dalziel et al., reported that NIH3T3 expression of K-Ras or H-Ras led to a 10-fold increase in ST6Gal1, although there were no significant changes in the expression of other sialyltransferases (Dalziel et al., 2004). This study further determined that ST6Gal1 elevation during fibroblast transformation by mutant H-Ras^V12G37^ principally occurred *via* the RalGEF signaling axis. Later, Seales et al. reported that during the differentiation of myeloid cells to monocytes/macrophages, ST6Gal1 expression was suppressed *via* the protein kinase C/Ras/ERK signaling cascade, indicating that the role of RAS in ST6Gal1 regulation is cell and context dependent (Seales et al., 2005a). Additional roles for downstream mediators of RAS signaling were demonstrated in melanoma, where BRAF mutations conferring constitutive activation are often present. Using 63 melanoma cell lines, ST6Gal1 was identified by Johansson et al. as a gene that was consistently upregulated with activating BRAF mutation (Johansson et al., 2007). Furthermore, in a Genetically Engineered Mouse Model (GEMM) of pancreatic cancer with RAS activation *via* expression of K-Ras^G12D^ under the control of p48Cre (Qian et al., 2009), the loss of ST6Gal1 resulted in normal acinar area and decreased fibrosis (Kurz et al., 2021). This led to delayed disease development and progression (Kurz et al., 2021). Corroborating these findings, in pancreatic organoids developed from mice expressing K-Ras^G12D^ under the control of Pdx1-Cre, knockdown of ST6Gal1 inhibited organoid growth (Chakraborty et al., 2022). Considering RAS mutations are observed in up to 93% of pancreatic cancer patients, the results of these studies strongly suggest ST6Gal1 as a downstream mediator of, and potential therapeutic target in, RAS-mediated pancreatic oncogenesis. These studies highlight the importance of ST6Gal1 in cancers with RAS activation, but the mechanisms through which RAS-mediated alterations in ST6Gal1 expression to promote malignant features still remain outstanding.

### PI3K/AKT signaling

The phosphatidylinositol 3-kinase (PI3K)/protein kinase B (AKT) signaling cascade, a known RAS effector cascade, influences many biologies important in cancer, including cell cycle regulation, proliferation and apoptosis (Jiang et al., 2020). This pathway is activated in a multitude of cancers and, in turn, leads to uncontrolled cell growth, migration, invasion and therapeutic resistance (Castellano and Downward, 2011; Jiang et al., 2020). While the impact of sialylation and/or ST6Gal1 on the PI3K/AKT pathway remain to be fully explored, recent data suggest PI3K/AKT signaling can be activated *via* elevation of ST6Gal1. The overexpression of ST6Gal1 in the colon carcinoma cell line SW480 increased adhesion to fibronectin and led to cell survival *via* activation of AKT as measured by levels of phospho-AKT (Lee et al., 2010). In the same study, targeting ST6Gal1 with siRNA in SW480 cells increased sensitivity to radiation induced cell death through a mechanism associated with decreased phospho-AKT. When the colon cancer cell line HCT116 was treated with nano-diamino-tetrac (NDAT), an antiproliferative/angiogenic agent, the protein expression of ST6Gal1 along with phospho-PI3K was diminished (Chang et al., 2018). However, another study in HCT116 cells reported that knockdown of ST6Gal1 had no effect on expression of AKT or phospho-AKT, and there were no changes in cell adhesion or proliferation (Meezan et al., 1969; Qian et al., 2009). In this report, decreased expression of ST6Gal1 reduced motility through decreased c-Met/STAT3. This hints towards a more nuanced function of ST6Gal1-AKT axis in colon cancer even though a pattern of ST6Gal1-mediated increases in PI3K/AKT signaling was often observed.

ST6Gal1 was also associated with changes in PI3K/AKT signaling in hepatocellular carcinoma (HCC). The overexpression of ST6Gal1 in the less invasive HCC cell line MHCC97L, increased invasion along with expression of PI3K p110α (the catalytic subunit of PI3K) and phospho-AKT (Zhao et al., 2014). In the same study, converse experiments demonstrated that targeting ST6Gal1 in the metastatic HCC cell line MHCC97H decreased invasion as well as expression of PI3K p110α and phospho-AKT (Zhao et al., 2014). In a tissue microarray of HCC, ST6Gal1 expression correlated with worse prognosis and caveolin-1 expression. When caveolin-1 expression was silenced in MHCC97H cells, the expression of ST6Gal1, phospho-PI3K, phospho-AKT and phospho-mTOR also diminished simultaneously (Chen et al., 2021). Consistent with the previous studies, HepG2 HCC cells treated with exosomes isolated from MHCC97H cells had elevated phospho-AKT which promoted an invasive and proliferative phenotype (Wang et al., 2021). In these studies, phospho-GSK3β was also increased, and the PI3K/AKT/GSK3β signaling axis is established to promote cell survival (Romorini et al., 2016). In converse experiments using exosomes isolated from ST6Gal1 KD MHCC97H cells, the expressions of phospho-AKT and phospho-GSK3β were suppressed. In addition to ST6Gal1-mediated regulation of PI3K/AKT signaling, additional data suggests the potential for PI3K/AKT signaling to also regulate ST6Gal1. When the HCC line, Huh7, was treated with the AKT inhibitor MK2206 and the PI3K inhibitor LY294002, protein expression of ST6Gal1 was deceased. Overall, these data demonstrate the importance of ST6Gal1 in the regulation of PI3K/AKT signaling while also suggesting a possible feedback loop in HCC.

Beyond colon and liver cancers, ST6Gal1-mediated activation of the PI3K/AKT axis has also been reported in fibroblasts as well as other cancer types. For example, ST6Gal1 enhanced PI3K/AKT signaling in a monkey kidney fibroblast cell line, Cos7, to increase invasion and proliferation (Rao et al., 2022). This study linked the activation of PI3K/AKT signaling to ST6Gal1-mediated sialylation of EGFR (Rao et al., 2022). Considering the importance of amplifications of EGFR in cancer, this mechanism of ST6Gal1 action may also contribute to oncogenesis and/or tumor progression. In ovarian (Isaji et al., 2014) and breast cancer (Lu et al., 2014), ST6Gal1 mediated activation of the PI3K/AKT pathway was reported to promote invasion and EMT, respectively. Furthermore, in the prostate cancer cell lines, PC-3 and DU145, cell proliferation along with PI3K/ALK signaling was reduced when the ST6Gal1 gene was silenced (Wei et al., 2016). In gastric cancer, ST6Gal1 elevated phosphorylation of AKT in association with the promotion of resistance against trastuzumab (Liu et al., 2018). In this study, the activation of PI3K/AKT was due to ST6Gal1-mediated α2,6 sialylation of Human Epidermal Growth Factor Receptor 2 (HER2) (Liu et al., 2018), a breast cancer biomarker. In the BxPC3 pancreatic cancer line and the ovarian cancer cell line OV4, ST6Gal1 was also identified to provide protection against serum starvation *via* AKT signaling (Britain et al., 2017). As these data demonstrate, there are clear indications that PI3K/AKT signaling enrichment due to ST6Gal1 impacts a plethora of malignant phototypes like proliferation, growth, invasion and metastasis; however, the precise means by which these systemic changes are conveyed remain poorly understood with only a few receptors that increase AKT signaling identified as targets for ST6Gal1-mediated α2,6 sialylation.

### WNT signaling

The Wnt pathway regulates development and stem cell maintenance through canonical (dependent on β-catenin stabilization and translocation into the nucleus for downstream signal transduction) and non-canonical signals (β-catenin independent) (Zhan et al., 2017; Wen et al., 2020). Among a handful of investigations on the impact of ST6Gal1 on WNT signaling, most have focused on the canonical pathway. Elevated Wnt signaling, including *via* increased expression of regulators like WNT3A and β-catenin, was identified in RNA-sequencing analysis of metastatic subclones of pancreatic cancer cell lines S2-LM7AA and S2-013 that expressed high levels of ST6Gal1 (Britain et al., 2021). This finding was recently corroborated with RNA-sequencing analysis of the pancreas from GEMM models with pancreas-specific ST6Gal1 overexpression (Chakraborty et al., 2022): these data showed elevated stem cell-related pathways including Wnt (Chakraborty et al., 2022). Furthermore, elevated levels of ST6Gal1 in the colorectal cancer cell lines Caco-2 and SW48 were associated with increased expression of WNT3a and β-catenin as well as fluorouracil (5-FU) resistance (Cui et al., 2018). As mentioned earlier, decreased expression of ST6Gal1 in prostate cancer models results in diminished activation of a PI3K/AKT/GSK-3β signaling axis, leading to diminished β-catenin (Wei et al., 2016). In addition to these data suggesting ST6Gal1-mediated regulation of WNT signaling, additional information indicates that Wnt regulates ST6Gal1 expression. In organoids derived from gastric cancer, ST6Gal1 expression depended upon stem cell maintenance factors with an important role for Wnt (Alexander et al., 2020). Modulation of cancer stem cell maintenance by the Wnt pathway is well recognized (Espada et al., 2009; Duchartre et al., 2017; Zhan et al., 2017; Patel et al., 2019), as is the promotion of stem cell maintenance by ST6Gal1 (Christie et al., 2008; Zhuo and Bellis, 2011; Schultz et al., 2013; Swindall et al., 2013; Wang et al., 2015; Chen et al., 2016; Schultz et al., 2016; Wei et al., 2016; Zhang et al., 2016; Britain et al., 2018; Chakraborty et al., 2018; Cui et al., 2018; Dorsett et al., 2019; Alexander et al., 2020; Verge et al., 2020) in myriad of neoplasms. Given the evidence of differential regulation of the Wnt pathway in ST6Gal1-modulated systems, and the importance of both Wnt and ST6Gal1 in cancer stem cell maintenance, it is imperative to understand the upstream and downstream mechanisms though which this signaling axis acts.

### TGF-β signaling

Transforming Growth Factor-Beta (TGF-β) is a cytokine known to play a dual role in cancer; it often acts as a tumor suppressor in the earlier stages of disease due to its ability to inhibit cell proliferation while later promoting tumor progression in association with increased angiogenesis, EMT, immunosuppression and invasion (Bierie and Moses, 2006; Du et al., 2015; Hao et al., 2019). Therefore, reports of TGF-β signaling in relation to ST6Gal1 reflect these dynamic functions. For example, a study by Du et al. showed that TGF-β-induced EMT is regulated by sialylation, including that mediated by ST6Gal1, in HaCaT, MDCK and A549 cells. After a short 4-h treatment with TGF-β, ST6Gal1 and other sialyltransferases were downregulated, followed by gradual recovery of ST6Gal1 over time and upregulation at 24 h post treatment (Du et al., 2015). Additional studies confirmed that ST6Gal1 expression was elevated by TGF-β stimulation in other cell types, including the mouse epithelial cell line GE11 where transcriptional upregulation of ST6Gal1 involved SP1 (Lu et al., 2014). In this study that also included MDA-MB-231 breast cancer cells, knockdown of ST6Gal1 resulted in decreased EMT characteristics in association with PI3K/AKT signaling (rather than Smads) and preservation of E-cadherin (Lu et al., 2014). ST6Gal1 was also increased by TGF-β in THESCs endometrial fibroblasts, 12Z immortalized endometrial cells and Ishikawa endometrial cells (Choi et al., 2018). As early-stage endometriosis is defined by focal adhesion of endometrial tissue to the peritoneum, it was interesting to note that knockdown of ST6Gal1 in the Ishikawa endometrial adenocarcinoma cell line reduced TGF-β1-induced adhesion (Choi et al., 2018). While these data suggest an overall trend of TGF-β-mediated upregulation of ST6Gal1, it is important to recognize that not all cells have been reported to increase ST6Gal1 levels in response to TGF-β treatment: in HCV29 bladder epithelial cells, TGF-β decreased expression of ST6Gal1 (Guo et al., 2014). Whether this difference in TGF-β-mediated regulation of ST6Gal1 is cell type and/or time course dependent needs further investigation.

In addition to direct roles in neoplastic cells, ST6Gal1 and TGF-β signaling interactions can impact the tumor microenvironment. TGF-β1 is a well-established immunosuppressive cytokine (Yoshimura and Muto, 2011; Sanjabi et al., 2017; Batlle and Massague, 2019), and ST6Gal1 may enhance TGF-β1 secretion. In a study of HCC probing immune escape of cancer cells *via* modulation of T cell function (Wang et al., 2019), cells exhibiting upregulated ST6Gal1 were co-cultured with cytotoxic CD8^+^ T-cells resulting in increased secretion of TGF-β1 by the T-cells. These analyses indicate that, along with cell and context dependent functions in cancer EMT and metastasis, ST6Gal1-modulated expression of TGF-β has potential roles in immunosuppression.

### Integrins, cadherins, Ig-CAMs, and selectins and roles in cell adhesion

In cancer, interactions between cells as well as cell to matrix interactions play critical roles in malignant processes such as stem cell maintenance, cell fate and differentiation, inflammatory response, angiogenesis, migration, EMT, cancer progression and metastasis (Cavallaro and Dejana, 2011; Windisch et al., 2019; Janiszewska et al., 2020). These functions are orchestrated by cell adhesion molecules spanning four families of 1) Integrins (α2β1, α5/β1, αL/β2); 2) Cadherins (E-cad, P-cad, N-cad); 3) Ig-CAMs (VCAM, NCAM, ICAM, Nectins, Necl); and 4) Selectins (E-selectin, P-selectin, L-selectin). Roles of ST6Gal1 in promoting EMT and metastasis *via* differential expression of several adhesion molecules have been established. Recently, an OMICS network analysis investigating adhesion proteins revealed that sialylation of adhesion molecules, specifically by ST6Gal1, plays a vital role in cancer cell EMT, migration and invasion (Bauer et al., 2020). Indeed, the importance of integrin sialylation by ST6Gal1 in imparting the aforementioned neoplastic characteristics is one of the most well described ST6Gal1-regulated pathways.

The β1 subunit of integrin combines with α2/α4/α5 subunits (among others) to form connections with the extracellular matrix that affect the survival, migration, and metastasis of cancer cells (Pan et al., 2018). There is ample evidence that underscores ST6Gal1-mediated expression and α2,6 sialylation of β1-integrin in mammary cancer (Hedlund et al., 2008; Lu et al., 2014), HCC (Han et al., 2018), gliomas (Kroes and Moskal, 2016), ovarian cancer (Christie et al., 2008; Choi et al., 2018), CRC (Seales et al., 2005b) and even in adipogenesis (Kaburagi et al., 2017). α2,6 sialylation of the β1 complex was also reported to affect binding to receptors: α5β1 to fibronectin (Pretzlaff et al., 2000; Semel et al., 2002; Seales et al., 2005a), α4β1 to VCAM-1 (Woodard-Grice et al., 2008), α3β1 to laminin (Pochec et al., 2003) and α1β1 (Seales et al., 2003) as well as α2β1 to collagen (Shaikh et al., 2008). In the colon adenocarcinoma cell line SW480, Lee et al. employed both overexpression and knockdown models to further establish that ST6Gal1 enhances the stability of β1-integrin (Lee et al., 2008). In the same cancer model, ST6Gal1 activated β1-integrin, further enhancing attachment to collagen and laminin: this resulted in heightened motility and highlighted the importance of ST6Gal1-regulated β1 integrin in disease progression and migration (Seales et al., 2005b; Chiricolo et al., 2006). A similar pro-migratory role was determined in HD3 colon epithelial cells in which Ras regulates ST6Gal1 expression and thereby addition of α2 sialic acid in β1, but not in β3 or β5, integrins: removal of α2 sialic acid from β1 integrin inhibited collagen binding and decreased migration and invasion (Seales et al., 2003; Shaikh et al., 2008). An additional report using SW480 cells confirmed overexpression of ST6Gal1 increased sialylation of β1 integrin and increased migration, while also suggesting β1 integrin-independent roles for soluble ST6Gal1 in migration (Lee et al., 2012). In a study with colon adenocarcinoma cells lacking α2,6 sialylation (SW48), ST6Gal1 expression was found to decrease adhesion to galectin-3 coated plates: interestingly, unsialylated β1 integrin bound to galectin-3 and promoted apoptosis, but ST6Gal1 expression and α2,6 sialylation of β1 integrin protected cells from galectin-3 induced apoptosis (Zhuo et al., 2008). ST6Gal1-mediated hypersialylation of β1 integrin in colon cancer cells also resulted in enhanced fibronectin binding that promoted survival *via* activation of a downstream cascade involving paxillin and AKT (Lee et al., 2010). Together, these data suggest sialylation of β1 integrin is critical for modulating extracellular matrix associations that mediate pro-tumorigenic colon cancer biologies including attachment, survival, and migration. Similar to these studies in colon cancer models, cell adhesion in the HCC cell line H22 *via* α5β1 integrin required α2,6 sialylation (Yu et al., 2013) and ST6Gal1-mediated hypersialylation of integrin β1 resulting in increased attachment to collagen I was determined in ovarian carcinoma (Christie et al., 2008). In the ovarian cancer models, the ST6Gal1/integrin-mediated signals imparted an invasive phenotype. While a pro-migratory and invasive phenotype of ST6Gal1 mediated by β1 sialylation is often determined, this is not always the case: ST6Gal1 knockdown increased metastasis as well as expression of integrin α3β1 in the metastatic CRC cell line SW620 (Jung et al., 2016).

In addition to roles in attachment and invasion, ST6Gal1/integrin-mediated signals have been associated with changes in cell fate impacting survival and differentiation. ST6Gal1 sialylation of β1 integrin in colon cancer also enhanced radio-resistance (Lee et al., 2008). Furthermore, in the ovarian cancer line OVCAR4, ST6Gal1-mediated α2,6 sialylation was essential for integrin α2-dependent cancer cell survival (Huang et al., 2021). In an *in vivo* model of mice mammary carcinoma, decreased cancer differentiation was observed with elevated ST6Gal1 and β1-integrin (Hedlund et al., 2008).

There are additional roles for ST6Gal1-mediated regulation of β1-integrin and cadherins in oncogenic transformation from epithelial (low oncogenic potential) to mesenchymal (high oncogenic potential) phenotypes. To indicate epithelial to mesenchymal transition (EMT), elevation of N-cadherin and downregulation E-cadherin are commonly used biomarkers. In the MG-63 osteosarcoma cell line, targeting ST6Gal1 decreased expression of N-cadherin, while increasing the expression of E-cadherin (Meng et al., 2015). As mentioned earlier, Lu et al. reported that during TGF-β-mediated EMT in breast cancer, expression of ST6Gal1 was elevated and, in turn, was inversely proportional to E-cadherin and directly proportional β1-Integrin levels (Lu et al., 2014). In a human lung adenocarcinoma model with cisplatin resistance, migratory capacity increased with ST6Gal1 and N-cadherin expression while E-cadherin expression was reduced (da Fonseca et al., 2022). A similar, elevated expression of N-cadherin with high ST6Gal1 was associated with invasive characteristics in pancreatic cancer (Britain et al., 2021) and HCC (Chen et al., 2021). In ovarian cancer, regulated by the P120 canonical pathway, adhesion of cancer cells to the peritoneal mesothelium stimulates ST6Gal1 expression facilitating hypersialylation of β1-integrin and elevation of P-cadherin (Britain et al., 2021). A comparable investigation in ovarian cancer showed that elevation of ST6Gal1 led to P-cadherin enrichment that resulted in upregulation of β1-integrin and drove metastasis *via* p70 S6 kinase activity (Britain et al., 2021). Together, these data link ST6Gal1, integrins, and cadherin to EMT and invasion, with β1-integrin being a direct target for ST6Gal-mediated sialylation.

ST6Gal1 also modulates angiogenesis *via* regulation of integrins and PECAM. PECAM is an important endothelial adhesion molecule with roles in cell survival and signal mechanotransduction (Kitazume et al., 2010). PECAM is a direct substrate of ST6Gal1 in mouse endothelial cells and α2,6 sialylation is crucial for its cell surface retention, PECAM-PECAM interaction (clustering), downstream signaling and anti-apoptotic function (Kitazume et al., 2010; Kitazume et al., 2014; Lee et al., 2014). In node-negative breast cancer patients, higher ST6Gal1 expression was associated with high E-selectin expression and lower survival (Hebbar et al., 2003). In terms of classical functions in angiogenesis, VEGFR2 and integrin β3 are both α2,6 sialylated and interact with PECAM. When ST6Gal1 knockout mice were injected with lung carcinoma cells, there was reduced PECAM surface stabilization and the PECAM-VEGFR2 interaction was compromised: this led to apoptosis in endothelial cells and inhibition of tumor angiogenesis (Imamaki et al., 2018). Another essential cell adhesion molecule, ICAM, is known to be downregulated in metastatic CRC (Maeda et al., 2002; Tachimori et al., 2005). In CRC, ST6Gal1 expression is higher in tumor compared to normal tissue and interestingly, lower expression is observed in metastatic (stage III and IV) tumors compared to the non-metastatic ones (stage I and II) (Zhang et al., 2017). A study in CRC using SW480 and SW620 lines showed that stabilization of ICAM by ST6Gal1 led to decreased metastasis (Zhou et al., 2019).

Although, there is still much to be explored, there have been some reports on the effects of ST6Gal1 on adhesion molecules in the context of the immune system. In U937 and THP-1 myeloid cell lines during differentiation with phorbol 12-myristate 13-acetate, ST6Gal1 expression decreased and, consequently, β1 integrin was hyposialylated: there was enhanced binding to fibronectin (Semel et al., 2002) that was regulated by the PKC/Ras/ERK pathway (Seales et al., 2005a). Another investigation of cell junctions in monocytes revealed that treatment with the proinflammatory cytokine TNF-α resulted in a concomitant decrease of ST6Gal1 expression and VE-cadherin α2,6 sialylation (Deng et al., 2017). Vascular Cell Adhesion Molecule (VCAM) interacts with α4β1 integrin to mediate leukocyte adhesion. In flow conditions, a decrease in VCAM1-mediated adhesion was reported in association with reduced ST6Gal1. ST6Gal1 was also reported to remove α4β1-dependent VCAM1 binding in monocytes (Woodard-Grice et al., 2008). However, the immunomodulatory impacts of ST6Gal1 in general and in the context of cancer remain to be further explored: the importance of these studies will only increase with expanded use of immunotherapies.

As evident from these data, ST6Gal1 function is crucial for the stability, signal transduction and function of adhesion molecules. Even though, among the adhesion molecules, the relationship of ST6Gal1 to integrins has received the most attention, we are still far from understanding the entirety of the upstream regulators and downstream signaling cascades of ST6Gal1-integrin association. Even fewer studies have been conducted on cadherins, which are important in cell-to-cell adhesion, or on selectins, which are important for blood cell-to-endothelial cell adhesion. Only a handful of studies have focused on Ig-CAMs, including one pertaining to Necl in lung adenocarcinoma, where ST6Gal1 was identified as a target of mir-199a leading to reduction of Necl-2 sialylation (Minami et al., 2013). Thus, there are many aspects of ST6Gal1 and sialylation-mediated regulation of adhesion molecule signaling that remain to be fully elucidated.

### TNF family of death receptors and cytokines

The Tumor Necrosis Factor (TNF) superfamily is a group of ligands and receptors that include death receptors like TNFR1, FAS (CD95/APO-1), DR3 (TRAMP, APO-3), DR4 (TRAIL-R1), DR5 (TRAIL-R2/APO-2/KILLER), and DR6. While ligand stimulation of TNFR1 by TNF often results in gene activation, stimulation of FAS by FasL, or DR4 and DR5 by TRAIL, often leads to apoptotic signaling cascades. The TNFR family of DRs have been implicated in neoplastic processes by impacting inflammation and tumor survival (Takeda et al., 2007; Walczak, 2013; Dostert et al., 2019). Contrary to its well established pro-apoptotic function, FAS also imparts cell survival and pro-proliferative characteristics in a multitude of models (O'Connell et al., 1998; Lamboley et al., 2002; Natoli et al., 1995; Landowski et al., 1997; Baldwin et al., 1999; Chen et al., 2012; Yuan et al., 2011; Nijkamp et al., 2010; Lai et al., 2010; Ametller et al., 2010; Peter et al., 2007). Interestingly, Lee et al., reported that while apoptosis induction was mediated by receptor internalization, Fas localized in plasma membrane imparted pro-survival signals (Lee et al., 2006).

Sialylation of Fas has been shown to mask its apoptotic function (Peter et al., 1995; Suzuki, et al, 2003; Keppler et al., 1999). More specifically, sialylation by ST6Gal1 of Fas hindered subsequent death complex formation and prevented internalization of Fas receptor, abrogating Fas-mediated apoptosis in colon cancer cells (Swindall and Bellis, 2011; Swindall et al., 2013). Similarly, ST6Gal1 is reported to directly α2,6 sialylate TNFR1 and block the TNFα-induced apoptotic pathway in macrophages (Liu et al., 2011a), rectal cancer (Smithson et al., 2022), and pancreatic and ovarian cancer (Holdbrooks et al., 2018). This phenotype of apoptosis inhibition was caused by prevention of TNFR1 internalization with α2,6 sialylation (Holdbrooks et al., 2018). Intriguingly, while TNFR1 sialylation by ST6Gal1 inhibited the apoptotic arm of this signaling cascade, TNF-induced signaling *via* NF-κB and AKT pathways was enhanced. Furthermore, these findings were corroborated in a gastric organoid model, where overexpression of ST6Gal1 led to increases in surface TNFR1 expression: this increase protected cells against apoptosis as a result of TNFR1 α2,6 sialylation, which led to reduced receptor internalization and degradation (Alexander et al., 2020). Studies in rectal cancer (Smithson et al., 2022) and pancreatic ductal adenocarcinoma models have also reported that ST6Gal1 mediates chemoresistance *via* evading apoptosis through a mechanism that potentially involves TNFR1 sialylation (Chakraborty et al., 2018). Thus, signaling of the TNF superfamily in cancer is often modulated by ST6Gal1-mediated sialylation to avoid cell death and thereby promote tumor growth.

### EGFR

Epidermal Growth Factor Receptor (EGFR) is a receptor tyrosine kinase activated by EGF ligand that is highly implicated in neoplastic mechanisms including invasion, metastasis, therapy resistance and angiogenesis (Henson and Gibson, 2006; De Luca et al., 2008; Chong and Janne, 2013; Sasaki et al., 2013; Sigismund et al., 2018; Uribe et al., 2021). Liu et al., determined that sialylation of EGFR affects its dimerization and potentially downstream signaling (Liu et al., 2011b). Since then, ST6Gal1-mediated activation of EGFR has been identified to influence survival, proliferation, apoptosis evasion, chemotherapy resistance, invasion and metastasis in a host of cancers including colorectal cancer (Liu et al., 2011b; Chang et al., 2018; Rodrigues et al., 2021) ovarian cancer (Schultz et al., 2013; Britain et al., 2018; Rao et al., 2022), and pancreatic ductal adenocarcinoma (Chakraborty et al., 2018). Using the CRC line SW480, Park et al. reported that EGF-induced EGFR activation and downstream pro-growth and proliferation signaling was enhanced with ST6Gal1 expression. Further, ST6Gal1-mediated resistance to the EGFR kinase inhibitor, gefitinib, was confirmed: these data suggested a major impact of ST6Gal1 in EGFR regulation (Park et al., 2012). When gefitinib-resistant CRC was treated with the antiproliferative agent nano-diamino-tetrac (NDAT), proliferation was abrogated *via* inhibition of ST6Gal1 (Chang et al., 2018). These findings in drug resistant cells were augmented by a recent study concluding that EGFR is indeed α2,6 sialylated by ST6Gal1 in a glycosite specific fashion: this modification of EGFR was, in part, responsible for resistance to cetuximab induced cytotoxicity in CRC (Rodrigues et al., 2021). These reports were corroborated in an ovarian cancer model (OV4 and SKOV3 cell lines) which showed a direct correlation between EGFR activation and ST6Gal1 expression, further confirmed EGFR as substrate of ST6Gal1, and demonstrated that ST6Gal1-mediated sialylation of EGFR leads to gefitinib resistance (Britain et al., 2018). ST6Gal1-mediated EGFR regulation was recently shown to foster elevated integrin forces, inferring a role in migration (Rao et al., 2022). The same study revealed that elevated expression of ST6Gal1 led to sustained membrane retention of EGFR. In support of this notion, elevated expression of ST6Gal1 concurrently activated EGFR in the pancreatic cancer cell line Suit2, exhibiting higher invasion and elevated levels of mesenchymal markers (Britain et al., 2021). These findings emphasize the importance of ST6Gal1-mediated α2,6 sialylation of EGFR in its turnover, clustering, activation and downstream signaling to effectively dictate its impact in cancer phenotypes.

### VEGF

The binding of Vascular Endothelial Growth Factor (VEGF) to its receptors, VEGFR1 and VEGFR2, regulates angiogenesis. In tumors, VEGF and its receptors are known to promote the tumor vasculature and, in turn, increase cancer growth (Carmeliet, 2005; Hicklin and Ellis, 2005; Ellis and Hicklin, 2008; Goel and Mercurio, 2013; Apte et al., 2019). A plethora of reports have established the importance of glycosylation in neoplastic angiogenesis: in particular, VEGFR2 sialylation was critical for angiogenesis mediated by VEGF (Lynch et al., 2012; Croci et al., 2014a; Croci et al., 2014b; Croci and Rabinovich, 2014; Chandler et al., 2017; Chiodelli et al., 2017; Cheng and Oon, 2018). As mentioned earlier, in a mouse Lewis lung carcinoma, while α2,6-sialylated PECAM interacted with VEGFR2, loss of ST6Gal1 inhibited the interaction: this inhibition resulted in apoptosis and prevention of angiogenesis (Imamaki et al., 2018). Similarly, in an osteosarcoma model, loss of ST6Gal1 led to decreased VEGF expression (Meng et al., 2015). Utilizing ST6Gal1 null mice inoculated with B16-F0 melanoma tumors, it was revealed that α2,6 sialylation was high in tumors sensitive to anti-VEGF monoclonal antibody treatment, and ST6Gal1 knockout also led to protection against anti-VEGF treatment (Croci et al., 2014a). The relationship of ST6Gal1 to VEGF was also determined in non-small cell lung cancer cells in which ST6Gal1 downregulation caused Notch1 pathway disruption and subsequently decreased protein expression of VEGF along with MMP-2, MMP-7 and MMP9: this resulted in reduced proliferation, migration and invasion (Sartakhti et al, 2017). One of the factors capable of upregulating VEGF expression, leading to increased angiogenesis, is Hypoxia Inducible factor 1 (HIF1) (de Palma et al., 2017; Zhang et al., 2018a; Zhang et al., 2018b). A recent study in ovarian and pancreatic cancer demonstrated that cells propagated in hypoxia exhibited an upregulation in ST6Gal1 (Jones et al., 2018). Furthermore, overexpression of ST6Gal1 also elevated accumulation of HIF1. As hypoxia is a major driver of VEGF mediated angiogenesis, this suggests a potential signaling axis of ST6Gal1/HIF1/VEGF that contributes to tumor growth *via* angiogenesis.

## Discussion

For pathophysiology of cancer, the perturbations in post-translational modifications like glycosylation are as important as the changes in genetic or protein content. However, compared to other facets, our understanding of the mechanistic basis through which sialylation influences molecular cascades in neoplastic transformation is limited. How a particular sialyltransferase, like the pro-oncogenic protein ST6Gal1, impacts molecular signals to regulate pro-tumorigenic cellular behaviors also remains to be fully determined. While ST6Gal1 has received some attention recently, important questions still remain unresolved to distinguish ST6Gal1 as a cancer biomarker. These include:

1) What are the upstream regulators of ST6Gal1 in different cancers at different stages?
2) What are the direct targets and interactors of ST6Gal1?
3) What are the changes to α2,6 sialylated proteins (confirmation, clustering, turnover) elicited by ST6Gal1?
4) What kind of effect (activating, deactivating) results from α2,6 sialylation of specific proteins?
5) Can we fully elucidate downstream mechanisms and signaling cross talk mediated by ST6Gal1 to develop novel anti-cancer therapeutic strategies?
6) Can we fully understand the specific targets altered by ST6Gal1 that lead to particular hallmarks of cancer to develop novel biomarkers?

It is apparent that ST6Gal1 plays a central role in cancer pathobiology, and thus holds a great potential for anti-cancer therapeutics. Further elucidation of these mechanistic cellular cascades will assist in the process of developing effective therapeutic mechanisms and prognostic guidelines for cancer.
